# Integrated Analyses of DNA Methylation and Gene Expression of Rainbow Trout Muscle under Variable Ploidy and Muscle Atrophy Conditions

**DOI:** 10.3390/genes13071151

**Published:** 2022-06-26

**Authors:** Mohamed Salem, Rafet Al-Tobasei, Ali Ali, Brett Kenney

**Affiliations:** 1 Department of Animal and Avian Sciences, University of Maryland, College Park, MD 20742, USA; areali@umd.edu; 2 Computational Science Program, Middle Tennessee State University, Murfreesboro, TN 37132, USA; rafet.al-tobasei@mtsu.edu; 3 Division of Animal and Nutritional Sciences, West Virginia University, Morgantown, WV 26506, USA; bkenney@wvu.edu

**Keywords:** rainbow trout, epigenomics, DNA methylation, muscle, ploidy, gene expression

## Abstract

Rainbow trout, *Oncorhynchus mykiss*, is an important cool, freshwater aquaculture species used as a model for biological research. However, its genome reference has not been annotated for epigenetic markers affecting various biological processes, including muscle growth/atrophy. Increased energetic demands during gonadogenesis/reproduction provoke muscle atrophy in rainbow trout. We described DNA methylation and its associated gene expression in atrophying muscle by comparing gravid, diploid females to sterile, triploid females. Methyl Mini-seq and RNA-Seq were simultaneously used to characterize genome-wide DNA methylation and its association with gene expression in rainbow trout muscle. Genome-wide enrichment in the number of CpGs, accompanied by depleted methylation levels, was noticed around the gene transcription start site (TSS). Hypermethylation of CpG sites within ±1 kb on both sides of TSS (promoter and gene body) was weakly/moderately associated with reduced gene expression. Conversely, hypermethylation of the CpG sites in downstream regions of the gene body +2 to +10 kb was weakly associated with increased gene expression. Unlike mammalian genomes, rainbow trout gene promotors are poor in CpG islands, at <1% compared to 60%. No signs of genome-wide, differentially methylated (DM) CpGs were observed due to the polyploidy effect; only 1206 CpGs (0.03%) were differentially methylated, and these were primarily associated with muscle atrophy. Twenty-eight genes exhibited differential gene expression consistent with methylation levels of 31 DM CpGs. These 31 DM CpGs represent potential epigenetic markers of muscle atrophy in rainbow trout. The DM CpG-harboring genes are involved in apoptosis, epigenetic regulation, autophagy, collagen metabolism, cell membrane functions, and Homeobox proteins. Our study also identified genes explaining higher water content and modulated glycolysis previously shown as characteristic biochemical signs of rainbow trout muscle atrophy associated with sexual maturation. This study characterized DNA methylation in the rainbow trout genome and its correlation with gene expression. This work also identified novel epigenetic markers associated with muscle atrophy in fish/lower vertebrates.

## 1. Introduction

Rainbow trout is one of the most well-studied fish [1]; however, complete genome annotation of it is still under development [2,3]. Availability of a genome sequence reference with complete annotation is essential for genomics- and epigenomics-based animal selection for aquaculture applications. Additionally, a completely annotated genome sequence will facilitate functional genomics and proteomics approaches for rainbow trout research and establish methods for comparative epigenomic analysis in a highly complex and duplicated genome [1]. Little is known about the methylome of fish, particularly the non-model species such as rainbow trout. A minimal number of DNA methylation studies have been conducted on rainbow trout, with almost no focus on annotation of the genome reference or integrated DNA methylation to gene expression [4,5]. Identifying the methylated DNA, epigenetic tags are essential for functional annotation of the rainbow trout genome.

Methylation of DNA cytosine is a critical epigenetic modification necessary for many vital biological functions, including cell differentiation, organismal development, epigenomic imprinting, and chromosome stability [6,7]. More than 99% of DNA methylation occurs in human somatic cells in a CpG context, while non-CpG methylation accounts for 25% in stem cells [8]. About 98% of methylated Cs in pufferfish was in the CpG context [9].

Despite many studies, the exact role of DNA methylation in regulating gene expression is still far from understood. The reported relationship between DNA methylation and gene expression varies between different classes of eukaryotes and may or may not be tissue-type specific [10]. For a long time, hyper-methylation of the gene promoter has been shown to regulate gene expression negatively by recruiting or blocking the binding of transcription factors or repressors to the promoter [6,7,11]. However, recent studies contradicted this notion and suggested enhancement of gene expression due to promoter hyper-methylation [10,12]. 

The muscle makes up about 50% of the fish body and is the most significant edible part of fish. However, few studies have targeted DNA methylation in fish muscle atrophy. 17β-Estradiol treatment increased non-CpG methylation of the MyoD gene exon-1 of the rainbow trout without affecting CpG sites. The study suggested a mechanism involving DNA methylation by which E2 reduces MyoD gene expression and decreases muscle growth [13]. In mammals, the exact mechanism of DNA methylation in muscle atrophy is still not well described. Loss of de novo DNA methylation in Dnmt3a knock-out mice caused decreased muscle mass, impaired muscle regeneration, and proliferation [14]. Muscle denervation causes reduced expression of DNMT3a and hypomethylation in the fibroblast growth factor-inducible-14 (Fn14) gene promoter. On the other hand, increased expression of DNMT3a reduces the expression of Fn14 and causes muscle atrophy [15]. Variations in DNA methylation of ten genes were reported in cancer-induced muscle atrophy [16]. Genome-wide DNA hypermethylation was associated with aging in a human muscle study. Differentially methylated CpGs were more predominant in the gene body than promoters [17]. The study did not find a correlation between DNA methylation with differential expression and identified 500 differentially methylated sites that distinguish between aged and young individuals. Therefore, the current study aimed to increase our understanding of the role of DNA methylation in mechanisms controlling muscle atrophy, particularly in fish. 

The diploid versus triploid fish used in this study offers a unique opportunity to investigate the role of DNA methylation on global gene silencing/regulation due to the polyploidy effect associated with gene dosage. An increase in the number of genomic sets in polyploidy is usually accompanied by infertility, lower genome stability, and dosage compensation [18,19]. A study of rice showed that ploidy (diploid to haploid and diploid to triploid) affects CHH methylation frequency [20]. The autotetraploid fish genome of *Carassius auratus* exhibited a lower genome-wide DNA methylation level than diploid fish [21]. A small-scale study on brown trout detected only 408 methylation loci and no difference in methylation between tripods and diploid fish [22]. 

This study investigated the potential role of DNA methylation in gene expression of gravid female diploid fish with atrophying muscle compared to sterile triploid rainbow trout. The elevated energetic demands during gonadogenesis/reproduction provide a unique model for investigating the role of DNA methylation in muscle atrophy in rainbow trout. Previously, we used this model to identify molecular/metabolic pathways characteristic of muscle atrophy in fish, including transcriptomic changes of protein-coding genes, microRNAs and long noncoding RNAs, and variations at the proteomics level [23,24,25,26]. The study’s first objective was to characterize the genome-wide methylome of rainbow trout muscle. The study’s second objective was to investigate the relationship between DNA methylation and gene expression. This study provides a genome-wide DNA methylation profiling of the polyploidy effect on DNA methylation of salmonids.

## 2. Materials and Methods

### 2.1. Fish Population and Muscle Sampling

The samples used in this study were previously described in a previous study [26]. Mature fertile/gravid (diploid) and sterile (triploid) female rainbow trout (~500 g) were sampled from Flowing Springs Trout Farm (Delray, WV, USA) during the sexual maturation/spawning season. Fish were cultivated in two identical raceways supplied with spring water at a temperature 13 ± 3 °C. All fish were fed as much as desired through a commercial diet demand feeder (Zeigler Gold; Zeigler Bros., Gardeners, PA, USA). Muscle samples were collected, and the gonado-somatic index (GSI) was 15.8 ± 0.3 (*n* = 5) in gravid fish compared to 0.3 ± 0.2 (*n* = 5) in sterile fish. The muscle tissue of 8 fish (4 fertile and 4 sterile) was collected from the dorsal side, flash-frozen in liquid nitrogen, and stored at −80 °C until DNA and RNA extraction. The muscle phenotypic characteristics of gravid and sterile fish have been previously described [26]. Briefly, gravid fish had lower muscle/whole body weight (49.9% ± 6.7% vs. 62.6% ± 2.2%, *p* = 0.01), less muscle protein content (16.9% ± 0.7% vs. ~19.1% ± 0.7%, *p* = 0.01), and softer muscle (shear force 178 ± 19 g/g vs. 240 ± 18 g/g, *p* = 0.01). Conversely, atrophied muscle had a higher water content (80.3% ± 0.7% vs. 77.2% ± 0.6%) and pH (6.61 ± 0.03 vs. 6.41 ± 0.04).

### 2.2. RNA Sequencing and Analyses

The transcriptomics results have been previously published [26] and re-analyzed here using the Swanson rainbow trout genome reference NCBI Accession: PRJNA335610 in combination with new DNA methylation data. The total RNA was extracted from muscle using the TRIzol method (Invitrogen, Carlsbad, CA, USA). The concentrations of RNA were determined using a Qubit 2 Fluorometer with Invitrogen™ Qubit™ RNA High Sensitivity assay kit. RNA was treated with DNase, and RNA integrity was assessed using gel electrophoresis. RNA sequencing was conducted at the University of Illinois at Urbana-Champaign, Roy J. Carver Biotechnology Center. RNA sequencing libraries were prepared using Illumina TruSeq stranded total RNA with Ribo-Zero gold protocol according to the manufacturer’s recommendations (Illumina Inc., San Diego, CA, USA). One barcoded sequencing library was prepared from each fish, and equal amounts of all libraries were pooled and sequenced in a single lane (2 × 100 reads) of an Illumina HiSeq 2000 sequencing platform. RNA-Seq data are available through the Sequence Read Archive (SRA), NCBI accession: SRP131630. Read mapping to genome reference and identification of DE genes were performed using the CLC genomics workbench (Qiagen Inc., Redwood City, CA, USA). DE genes between gravid and sterile fish were identified using EDGE test (FDR-*p*-value < 0.05, fold change: >2 or <−2) as previously described [26].

### 2.3. Methyl-MiniSeq Sequencing and Analyses

Muscle samples were processed and analyzed using the Methyl-MiniSeq^®^ Service; genome-wide bisulfite sequencing was completed at Zymo Research (Irvine, CA, USA). DNA was extracted using Quick-DNA Plus Miniprep Kit. Five hundred nanograms of genomic DNA was digested first with 60 units of TaqαI followed by 30 units of MspI (NEB) and then purified with Zymo Research DNA Clean & Concentrator™-5. According to Illumina’s specified guidelines, fragments were ligated to pre-annealed adapters containing 5′-methylcytosine instead of cytosine. Adaptor-ligated fragments of 150–250 bp and 250–350 bp were retrieved from a 2.5% NuSieve 1:1 agarose gel using Zymoclean™ Gel DNA Recovery Kit. The EZ DNA Methylation-Lightning™ Kit was used for the bisulfite treatment. PCR was performed and the products were purified with DNA Clean & Concentrator™-5 for sequencing on an Illumina HiSeq. Sequence data are available through the Sequence Read Archive (SRA), NCBI accession: PRJNA431930.

Sequence reads from Methyl-MiniSeq libraries were identified using standard Illumina base-calling software. Raw FASTQ files were adapter- and quality-trimmed using TrimGalore 0.6.5. Filled-in nucleotides were also trimmed using TrimGalore 0.6.5. FastQC 0.11.8 was used to assess the effect of trimming and the overall quality distributions of the data. Reads with low quality (<20) were filtered out. Duplicate reads were removed using the deduplication function in Bismark. Alignment to the rainbow trout genome (https://www.ncbi.nlm.nih.gov/assembly/GCF_002163495.1/, accessed on 2 May 2022) was performed using Bismark 0.22.3 [27]. Using Bismark Methylation Extractor, methylated and unmethylated read totals for each CpG site were called. Sites with coverage of fewer than ten reads were not considered for subsequent analyses. The methylation level of the cytosines was calculated as the number of reads calling C, divided by the total number of reads calling C and T. Rainbow trout CpG islands were extracted from NCBI genome reference to annotate the methylation sites. The MethylKit R package was used to calculate the differential methylation between the diploid/gravid and triploid/sterile fish [28]. CpG sites with less than ten read depths or more than 99.9th percentile of coverage in each sample were filtered out to account for PCR bias. CpG sites exiting in a minimum of three samples of each group (3 out of 4) were kept for further analysis. CalculateDiffMeth function was used to determine the differential CpG site using logistic regression to calculate *p*-values [28]. Hyper- and Hypo-methylation were determined based on a q-value < 0.01 and a more than 25% methylation difference. JMP Pro^®^, Version 15. SAS Institute Inc., (Cary, NC, USA) was used to generate figures and statistical analyses to produce measures of association between DNA methylation percent and gene transcription expression deciles within each 1 kb or specific region, as mentioned in the results, flanking the TSS, using the correlation multivariate and nonparametric Spearman’s correlation functions. PROMO was used to identify putative transcription factor binding sites in DNA sequences [29].

## 3. Results and Discussion

### 3.1. CpG Dinucleotide Content in the Rainbow Trout Genome

The average number of sequence read pairs per sample analyzed in this study was 44,610,663. The average cytosine percentage in the CpGs context was 11.74 ± 1%, compared to 22.56 ± 0% in CHG and 65.69 ± 1% in CHH (where H corresponds to A, T, or C). The highest percentage of the methylated cytosines (62.18 ± 1%) was in the CpG context compared to only 1.09 ± 0% in CHG and 1.78 ± 0% in CHH contexts.

The highest percentage of the methylated cytosines (62.18%) was in the CpG context compared to only 1.09% in CHG and 1.78% in CHH contexts. The number of CpGs in the rainbow trout genome is 35,336,288. The CpG dinucleotide frequency in the genome is 1.83%, which is 3.4-fold less than the expected frequencies of the sixteen dinucleotides. Mammalian genomes have ~5-fold fewer CpG dinucleotides than expected [30], with 70–80% of the CpGs being methylated [31]. Spontaneous mutations of methylated C residues to T by deamination explain the CpG under-representation in genomes. On the other hand, unmethylated C mutates to U, which is quickly repaired [32]. 

Consistent with our data, 69.60% of mature tilapia muscle CpG cytosines are methylated compared to only 0.57% and 0.47% of the CHH and CHG context, respectively [33]. Similar results were reported in pufferfish and zebrafish, where 65–80%, 0.25–1%, and 0.34–1% of cytosines are methylated in CpG, CHG, and CHH, respectively [33,34]. In humans, on the other hand, almost all DNA methylation (99.98%) exists in CpG dinucleotides; non-CpG methylation accounts for 25% of cytosines in stem cells, though [8]. Mice also have about 74% methylated CpGs compared to about 0.6% non-methylated CpGs [33]. These data generally indicate conservation of CpG methylation in eukaryotic genomes. 

In this study, 3,161,570 cytosines in the CpG context were classified as genic (located within ±10 kb of the TSS) and were considered in the subsequent analysis. The rest of the data were intergenic. 

### 3.2. Non-Island Genic CpGs and Their Associated Gene Expression

**Figure 1:**
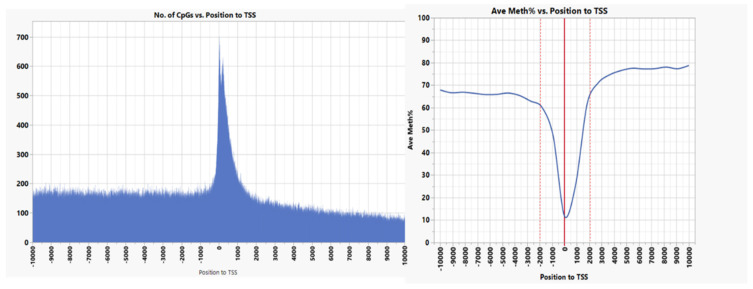
Number of CpGs per nucleotide (**left**) and their average methylation percentage relative to TSS (**right**). The average percent of CpGs methylation flanking TSS was 57.7% within ±10 kb and 31.6% within ±2 kb, reaching as low as 12% near the TSS.

Methyl Mini-seq revealed the existence of 2,916,293 non-island CpGs located within ±10 kb of TSS of 42,104 loci/genes. The overall CpGs density per nucleotide within the ±10 kb region was 145.8 CpGs/NT (Figure 1). However, the CpG density in the ±1 kb region flanking the TSS was 295.3 CpGs/NT, indicating enrichment of CpGs around TSS compared to other genic regions. 

Consistent with our observations, comparative epigenomic studies revealed conserved high CpG density around TSS. In fish, higher CpG density around TSS has been reported; however, the depletion of the CpG number, up-and down-stream of TSS, was less than that in mammals, perhaps due to lower GC content of fish genomes [35]. 

**Figure 2:**
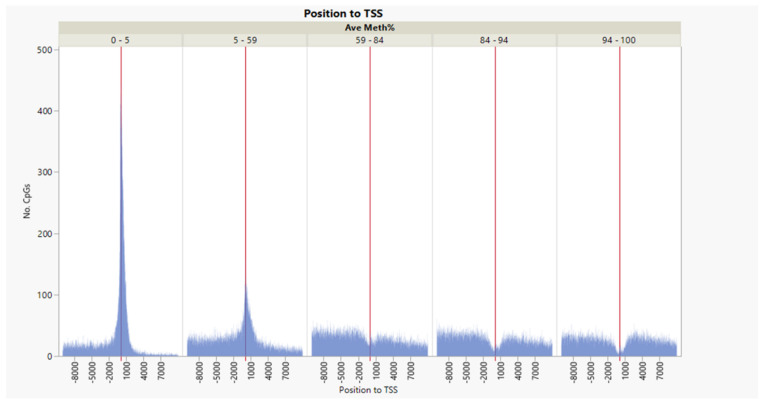
Number of CpGs per nucleotide relative to TSS at different intervals of average methylation percentages.

The average percent of CpGs methylation in ±10 kb flanking TSS was 57.7%; nevertheless, a sharp decline in DNA methylation was noticed in the ±2 kb region flanking the TSS. The average methylation percent of this region was 31.6% and reached as low as 12% near the TSS (Figure 1). Although CpGs increased near TSS, most of the CpGs were under-methylated. Figure 2 presents the number of CpGs per nucleotide near the TSS at different intervals of average methylation percentages.

Few studies investigated the genome-wide DNA methylation patterns in fish. A comparative epigenomics study including fish revealed that CpG density is strongly associated with the unmethylated state of DNA. Higher CpG density and lower methylation was reported around TSS in mouse and zebrafish livers [36]. Pufferfish promoters showed hypomethylated promoters in genes with intermediate CpG densities [35]. Studies explained that some proteins, such as CXXC-containing proteins and CG-rich binding transcription factors recruited by CpG-rich regions, maintain the unmethylated DNA state [35,37,38]. Another comparative study, including plants and animals, reported genome-wide high levels of CpGs methylation (~80%) in zebrafish larvae and mouse embryos [39]. A study on the tilapia genome showed a gradual decrease in CpG methylation level to 25% near the TSS compared to 75% in the gene body [33]. Low methylation levels on both sides of the TSS were reported in pufferfish with higher methylation levels in the gene body and downstream of genes [35].

Integrated analysis of Methyl Mini-seq and RNA-Seq showed a weak/moderate correlation between the average percentage of DNA methylation and gene transcription expression deciles calculated based on the expression ranking measured in RPKM (Reads Per Kilobase Million). However, this correlation was dependent on CpG position relative to TSS (*p* value < 0.0001). 

**Figure 3:**
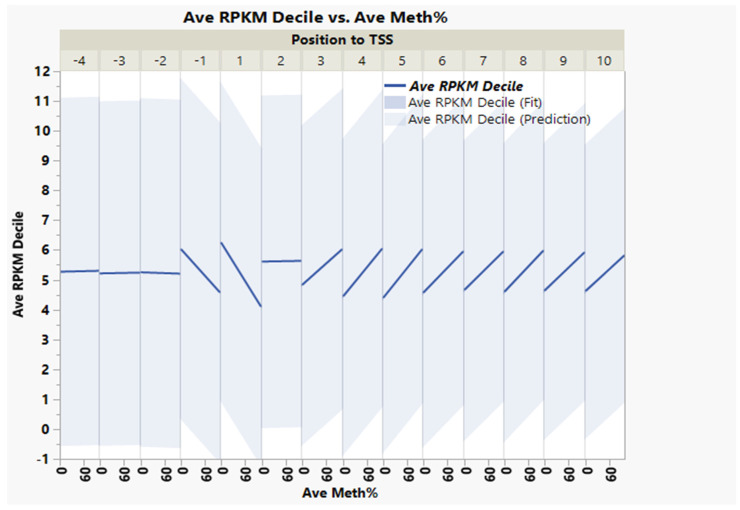
Inverse weak/moderate correlation between gene expression decile and DNA methylation within ±1 kb flanking TSS and positive correlation within the gene body’s +3 to +10 kb region.

**Figure 4:**
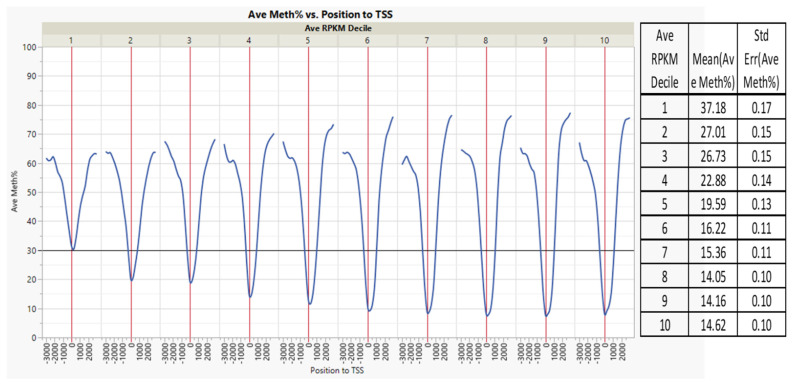
DNA methylation versus gene expression deciles. Inverse relationship between gene expression deciles (based on the increasing ranking of expression measured as RPKM) and gene methylation average percentages of CpGs within ±1 kb of TSS. The table shows the average gene methylation percentages and StdErr for each gene expression decile.

Within ±1 kb of the TSS, there was a negative correlation between the average DNA methylation percent and gene transcription expression deciles (Figure 3, correlation multivariate = −0.2046, *p* < 0.0001, nonparametric Spearman’s correlation = −0.2098, *p* < 0.0001). The average percentage DNA methylation within ±1 kb of the genes’ TSS at the first decile (lowest) of the expression range was 37.2%, compared to 14.6% in the 10th decile (highest) (Figure 4).

Conversely, within +3 to +10 kb in the gene body, there was a weak positive correlation between DNA methylation and gene expression (Figure 3, correlation multivariate = 0.1378, *p* < 0.0001, nonparametric Spearman’s correlation = 0.2000, *p* < 0.0001). 

Regarding the effect of CpG location on TSS and consistent with our data, fish and mammalian studies showed that gene expression is inversely correlated with DNA methylation in the first exon and the promoter [40,41]. A study of European sea bass showed a negative correlation between gene expression and the first intron, and the study speculated that the relationship might be due to transcription factor-binding motifs enrichment [40]. Conversely, a positive correlation has been demonstrated between gene expression and gene body methylation levels [42]. Moore et al. reported that the positive correlation between gene–body DNA methylation and gene expression is only in the dividing, not nondividing, cells [6]. In birds, however, CpG methylation at TSS and gene bodies of the great tit genome were negatively correlated with gene expression (Spearman’s rank correlation, Spearman’s rho < −0.23) [43]. The transcription factors’ sensitivity to DNA methylation can explain the TSS-flanking region methylation effect on gene expression [44]. However, the mechanism for explaining how gene body DNA methylation influences gene expression is still not clear. Guo et al. suggested a mechanistic link between gene transcription and DNA methylation at gene bodies. As gene transcription proceeds, H3K36me3 is deposited in gene bodies and helps recruit the DNA methyltransferase 3-DNMT3 PWWP domain [45]. The effects of DNA methylation on gene expression and splicing were also suggested, as reviewed in [30]. 

Few studies have investigated the relationship of DNA methylation to gene expression at the genome-wide level in fish. In tilapia as a general trend, a moderate negative correlation between CpG methylation in the gene promoter region (1000 bp upstream from TSS) and expression level was observed in male versus female fish (y = −0.28x + 43.7) [33]. Other studies have investigated DNA methylation relationships with a limited number of gene expressions. Atlantic Salmon challenged with high temperatures and hypoxia showed an inconsistent (positive and negative) correlation between CpG methylation levels and transcriptional changes of a few genes [46]. Another study on European sea bass detected global changes in DNA methylation caused by small ocean temperature increases. However, the study did not find a causal relationship between DNA methylation and gene expression [47]. Another study of sea bass looked at a few sex-relevant genes and reported a relationship between cyp19a1a methylation and its expression, but only under a methylation level of about 80%. However, the relationship was positive in males and negative in females [48]. In tongue sole fish, a correlation in expression of the dmrt1 gene and its DNA methylation during gonadal sex determination was reported after hatching [49]. The study showed that male-specific expression of dmrt1 during development was coordinated with the hypermethylation of the gene promoter in ovaries. Interestingly, this hypermethylation was reverted in genotypic females by high-temperature incubation, leading to the development of testes.

These studies suggest that DNA methylation is one of several epigenetic mechanisms regulating gene expression. Other mechanisms, such as histone modification or noncoding RNA, are also suggested to be necessary. A negative correlation between DNA methylation and histone H3K4me3 was observed across mammalian genomes [45]. Guo et al. showed that DNMT3A activity is induced when the histone H3K4 becomes unmethylated [45]. Additionally, if CpG is located in an enhancer or transcription factor- or repressor- binding site, CpG location may define the relationship between DNA methylation and gene expression, positive or negative [11,44].

### 3.3. CpGs Islands within or near Genes and Their Associated Gene Expression

Methyl Mini-seq revealed 245,287 CpGs within 6372 islands (CGI) in 6537 genes (±10 kb of TSS). Only 669 CGI were in the promoters (−2 kb of TSS) of 681 genes. The genes with promoter CGI comprise 10.4% of the total number of genes with CGI reported in this study (6537). This percentage of genes with promotor CGIs is a much smaller fraction than expected since the majority of the mammalian gene promoters, especially human housekeeping promoters, contain CGIs at an approximate frequency of 60% [50]. Moreover, promoters with CGIs are conserved between humans and mice [51]. To further explore our observation, we looked at the rainbow trout NCBI genome reference annotation and found only 349 genes with promotor CGIs within −1 kb of TSS, which is less than 1% of the genes in the genome. By contrast, 721 genes had CGIs in the first 1 kb of the gene body (discussed below, Figure 8). These data may indicate that rainbow trout gene promotors are generally CGI-poor relative to mammalian genomes. However, CGI density in fish genes, particularly promoters, has not been thoroughly investigated. Variation in the number and density of CGIs reported in 4 fish genomes was much broader than in other vertebrates, including mammals [52]. Computational CGI prediction models, based on mammalian species [53], may not accurately predict CGIs in cold-blooded vertebrates, shifting CGIs away from gene promoters [54]. In addition, the TSS of all the genes may not be completely annotated in the rainbow trout genome. Further studies are needed to explore CGI density in gene promotors of rainbow trout and other fish.

The average methylation level of promotor CGIs in this study was 55.73%. CGIs are usually hypomethylated to permit housekeeping and tissue-specific gene expression [50]. CpG-poor regions, on the other hand, are usually hypermethylated and involved in gene silencing [55].

**Figure 5:**
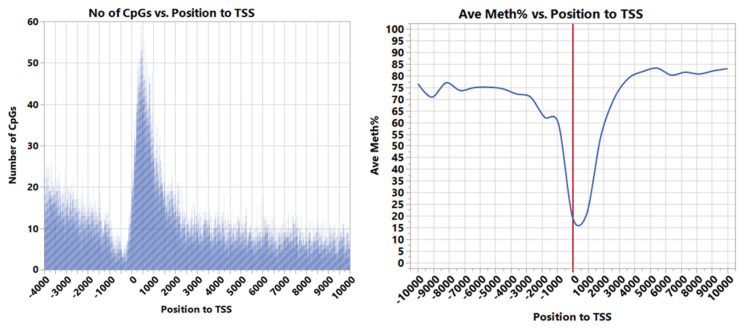
Number of CpGs located in CGI per nucleotide (**left**) and their average methylation percentage relative to gene TSS (**right**).

The CpG density within CGIs was 12.26 CpGs/NT, lower than that of the non-island CpGs (145.8 CpGs/NT) (Figure 5 vs. Figure 1). The CpG density −1 kb upstream of the TSS was 5.27 CpGs/NT, even lower than other genic regions, contrary to the sharp increase in CpG density +1 kb downstream of the TSS; 34.8 CpGs/NT, the highest density in the gene body.

Average percent methylation of CpGs located in CGIs was 63.20%, compared to 57.7% of non-island CpGs. However, similar to non-island CpGs, a steep decline in DNA methylation was noticed in the TSS-flanking regions (i.e., 1 kb in the gene promoter compared to +3 kb in the gene body). Average percent methylation of this region was 25.3% and declined to as low as 15% near the TSS (Figure 5). Beyond the −1 kb to +3 kb region flanking the TSS, a general trend of increasing DNA methylation levels was observed as the genomic window moved away from the TSS (Figure 5). Like the non-island CpGs, CpGs near TSS were predominantly unmethylated, although they were more abundant in number (data similar in trend to Figure 2 and not shown).

A comparative study, including plants and animals, reported high levels of CpG methylation (~80%) genome-wide in zebrafish larvae and mouse embryos; however, CGI methylation was low [39]. The study also reported slightly higher CpG methylation in the gene body and depletion of DNA methylation around TSS overlapping with CGI.

Combined analysis of the Methyl Mini-seq and RNA-Seq showed a trend similar to non-island CpGs in the relationship between average percent DNA methylation and gene transcription and expression deciles. This correlation varied according to CpG position relative to TSS (*p* value < 0.0001).

**Figure 6:**
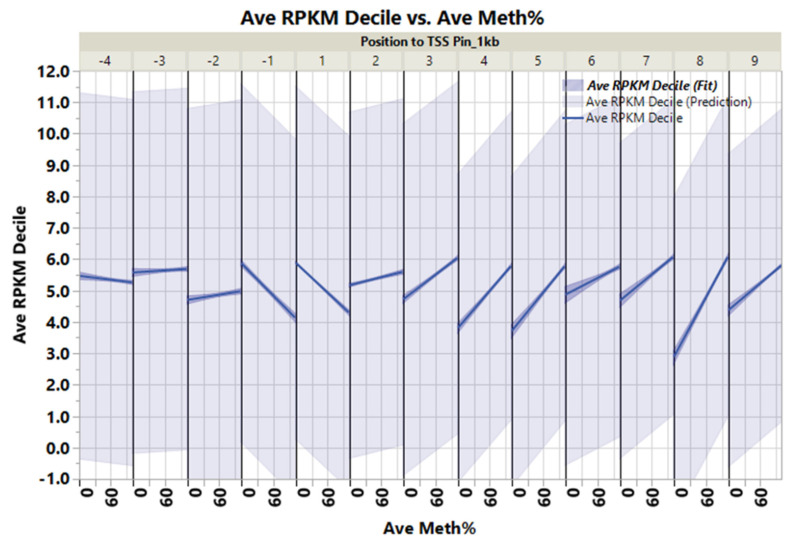
Inverse relationship between gene expression decile and DNA methylation in ±1 kb flanking TSS and positive correlation in +2 to +9 kb of the gene body.

**Figure 7:**
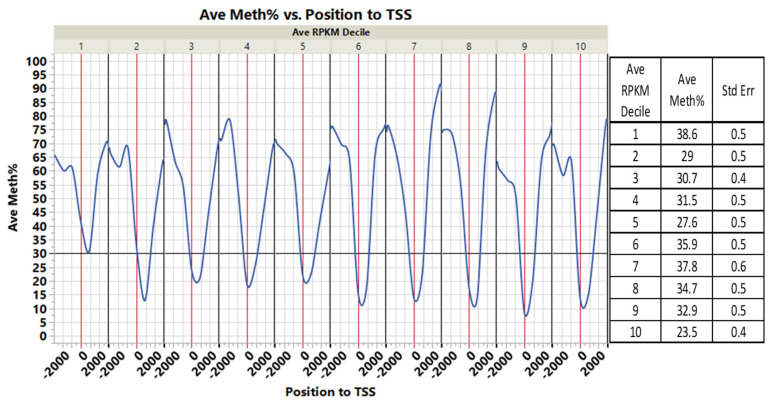
DNA methylation versus gene expression deciles. There is an inverse relationship between gene expression deciles (based on an increasing ranking of expression measured RPKM) and gene methylation average percentages of CGI within −2 kb and +2 kb of TSS. The table shows the average gene methylation percentages and StdErr for each gene expression decile.

Within ±1 kb of the TSS, there was a weak negative correlation between the average percentage of DNA methylation and gene transcription expression deciles (Figure 6, correlation multivariate = −0.1780, *p* < 0.0001, nonparametric Spearman’s correlation = −0.1954, *p* < 0.0001). This inverse relationship between gene expression decile and DNA methylation in the ±1 kb flanking TSS is further demonstrated in Figure 7. The average percent DNA methylation within ±1 kb of genes’ TSS at the first decile of the expression range was 38.6%, compared to 23.5% in the 10th decile (Figure 7).

In opposition and within +3 to +10 kb in the gene body, there was positive correlation between DNA methylation and gene expression in (Figure 6, correlation multivariate = 0.1546, *p* < 0.0001, nonparametric Spearman’s correlation = 0.2239, *p* < 0.0001).

Similar to our study, a negative correlation between CpG density and DNA methylation relative to TSS was reported in humans and other mammals, implying importance in essential molecular functions such as gene expression. However, precisely how the CGIs regulate gene expression is still unclear. Although TSS exist in about half of the CpG islands, those TSS often lack common promoter sequences [6,56]. DNA methylation’s role in regulating gene expression was suggested through interaction with nucleosome histone modification [6,57].

Studies concerning the relationship between CGI density, methylation, and gene expression are scarce in fish, especially non-model species. Zebrafish embryos exhibit low DNA methylation in CGIs [39]. Decreased de novo DNA methylation at the CpG island of the no-tail gene of zebrafish embryos is correlated with gene repression [58]. In gilthead sea bream, and although at a low level, DNA methylation was correlated with expression of the sirtuin-1 gene, a master regulator of metabolism. However, the involvement of other mechanisms in transcriptional regulation was suggested in the study [59].

In non-fish species, a recent study in chicken analyzed more than 3000 CGIs from 20 tissues and their association with gene expression. Scientists identified only 121 significantly correlated CGI-gene pairs, suggesting that alteration of CGI methylation rarely affects gene expression [60]. In a recent review, Bird, A. [50] claimed that embryonic gene transcriptional activity put methylation-free footprints on CGIs; methylation does not silence actively transcribed genes and only affects genes already silenced by other means. The involvement of DNA methylation in gene expression and its interaction with other epigenomics modifications warrants further investigation in fish.

**Figure 8:**
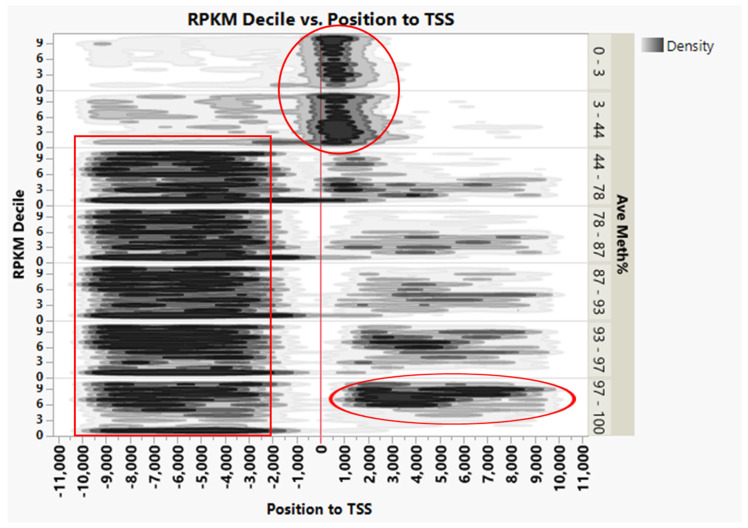
Density of CGIs in various genic regions and their relation to gene expression. Hypomethylation (0–44%) of CGIs was observed within 0 to +2 kb of TSS, regardless of gene expression level (top circle) and an increased percentage of the hypermethylated CGIs (>97%) in the highly expressed genes (right circle). Most CGIs at −2 to −10 upstream of genes are methylated (>44%), regardless of gene expression.

We noticed that most CGIs in the 0-to+2 kb region were hypomethylated (0–44%); however, this was not correlated with gene expression (Figure 8). A total of 1221 genes containing 1241 CGI (39,933 CpGs) showed these hypomethylated CGI marks. Interestingly, gene ontology annotation of the genes with these CGI marks revealed high enrichment of molecular functions relevant to DNA-binding and transcription regulation activity (Supplementary Tables S1 and S2). Examples of genes with essential functions in this list are 51 genes relevant to homeobox proteins, 24 forkhead box genes, 5 fibroblast growth factors, and 4 CCAAT/enhancer-binding protein genes. Homeobox gene methylation was reported in human lung cancer with reduced methylation in the CpG-rich region. This reduction was correlated with active histone H3 lysine-4 methylation chromatin mark in the HOXA region [61]. DNA methylation of some homeobox genes correlated with gene expression associated with muscle degradation (discussed below).

In addition, an increased percentage of hypermethylated CGIs (>97%) was noticed in the gene body (+2 to +10 kb) of the highly expressed genes (> 6 deciles). Moreover, most CGI in −2 to −10 upstream of genes were moderate- to hyper-methylated (>44%), regardless of gene expression (Figure 8).

**Figure 9:**
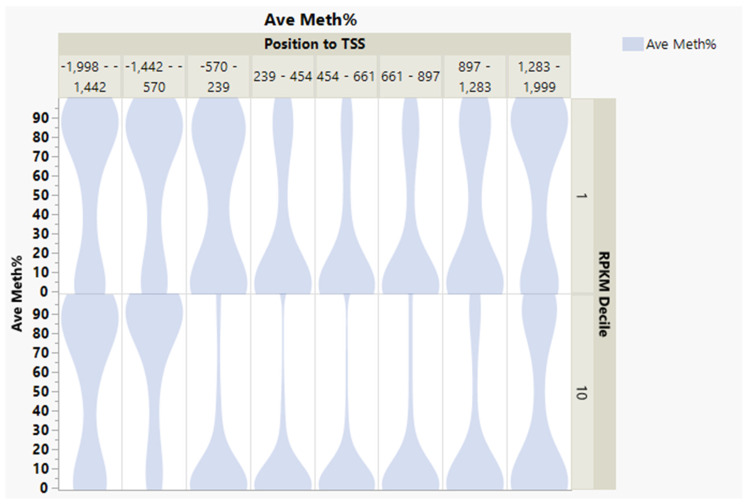
Silenced genes (RPKM Decile 1) in rainbow trout muscle have more hypermethylated CGIs in the promoter region compared to the most highly expressed genes (Decile 10).

A comparison between most-expressed genes (Decile 10) and silenced genes (Decile 1) revealed that silenced genes have more hypermethylated CGI around TSS −570 to 2 kb (Figure 9, Supplementary Table S4).

### 3.4. Differential DNA Methylation and Gene Expression between Diploid/Gravid and Triploid/Sterile Fish

#### 3.4.1. Differentially Methylated (DM) CpGs

We restricted our analyses of DM CpGs within −1 to +10 kb flanking the TSS of genes due to this region’s potential effect on gene expression. Between the diploid/gravid and triploid/sterile fish, there were 1206 DM CpGs located in 971 genes (Supplementary Table S3). This number of DM CpGs represents only 0.03% of the total CpGs (3,161,570) identified in this study, indicating no genome-wide effect of ploidy on DNA methylation, as hypothesized. Several genes had multiple CpGs, 11 had more than 4 DM CpGs, and 131 genes had 2 DM CpGs or more. Gene ontology (GO) enrichment analysis of the DM CpGs revealed enriched terms involved in regulating gene expression, metal transport, and cell adhesion under molecular functions and biological processes, as seen in Table 1.

Interestingly, our previous studies showed associations between cell adhesion genes and fillet softness in rainbow trout [62]. The gravid fish in this study had softer fillets than sterile fish [24]. Therefore, the identified DM CpGs could be used as epigenetic markers associated with muscle atrophy and quality. Further studies are needed to investigate the causal relationship between these DM CpGs and muscle atrophy.

#### 3.4.2. Integrated Analysis of Differential Gene Expression and DNA Methylation

Integrated analysis of differential gene expression and DNA methylation identified 31 DM CpGs associated with 28 differentially expressed genes involved in muscle atrophy, primarily (Table 2). The table shows that most DM CpGs were located in transcription factor binding sites. The list of transcription factors includes Pax 2/5/6 and 9, myogenin, and Hoxa5, which possess relevant muscle functions.

The genes spanning these 31 DM CpGs epigenetic markers were classified according to their functions in the following categories:

#### 3.4.3. Apoptosis and Epigenetic Regulation Genes

Apoptosis is a common mechanism of muscle atrophy associated with muscle denervation, muscular dystrophy, spinal cord injury, limb suspension, and immobilization [62]. This list includes two DM CpGs in the promotor of the programmed cell death protein-5 and one DM CpG in the apoptosis-enhancing nuclease. Moreover, we previously showed the involvement of apoptosis in rainbow trout muscle degeneration, using the same fish model as this study [23].

The epigenetic list includes a hypomethylated CpG in the gene body associated with the downregulated expression of the Histone-lysine N-methyl transferase *SMYD1* gene. *SMYD1* gene encodes a muscle-specific lysine methyltransferase with an essential role in fast-twitch muscle physiology and myofibril integrity. Mouse myofibers lacking the *SMYD1* are susceptible to atrophy [63].

There was also a hypermethylated CpG in the promotor associated with downregulated expression of the Cyclin-dependent kinase 2-associated protein-1 (*CDK2AP1*) gene. This gene impacts the cell cycle by negatively regulating the CDK2 activity and epigenetic regulation by participating in nucleosome remodeling and histone deacetylation [64].

Another gene involved in epigenetic regulation of muscle growth is ATP-dependent RNA helicase *DDX39A*, which had two hypomethylated CpGs in the gene body associated with reduced gene expression. Homozygous mutants of *DDX39A* exhibited cardiac and trunk muscle dystrophy in zebrafish due to early terminal differentiation of cardiomyocyte and myoblast; the mechanism of action of *DDX39A* involves obstructing mRNA splicing of members of the *KMT2* gene family [65].

#### 3.4.4. Autophagy, Glycolysis, Collagen Metabolism, Cell Membrane Functions Genes

The gene list for this category includes two genes relevant to autophagy named Autophagy-related protein-13 (*ATG13*), Sestrin-3, and 6-phosphofructo-2-kinase (*PFKFB3*), which is related to autophagy and glycolysis.

The autophagy gene pathway is vital for muscle energy homeostasis and bulk protein turnover. Dysregulation in the autophagy pathway can cause alterations in muscle growth, atrophy, and disorders, as reviewed in [66]. In vitro studies in rainbow trout showed that amino acid deprivation increases autophagosome formation and expression of autophagy-related genes [67]. Twenty-two genes involved in autophagy-related proteolysis were upregulated in rainbow trout muscle atrophy [26]. *ATG13* was upregulated in this study with hypomethylated CpG in the promoter regions. Interestingly, this CpG is located in the binding site of three transcription factors, PAX2, 9a/b.

Sestrins can induce autophagy that can cause cell death or have a cytoprotective effect depending on the metabolic and environmental context of the cell [68]. Sestrin can prevent muscle atrophy due to disuse and aging by coordinating anabolic and catabolic signals [69].

*PFKFB3* regulates glycolysis by modulating the level of fructose-2,6-bisphosphate. We previously reported that *PFKFB3* is upregulated in rainbow trout muscle atrophy [70]. *PFKFB4* was suggested to be a novel autophagy regulator through the suppression of oxidative stress [71]. Autophagy protects cells from severe conditions such as limitations in energy supplies and nutrients, and autophagy was suggested to be regulated by glycolysis in cancer cells [72]. Our previous studies showed reduced expression of genes involved in glucose use at transcription and proteomics levels [23,25], which is a common symptom of muscle atrophy shared with several mammalian models, including diabetes, fasting, cancer, renal failure, and muscle unload [73,74]. In this study, crosstalk between autophagy and glycolysis in atrophying muscle cells is logical because severe muscle atrophy occurs in response to the higher energetic demands of fish ovarian development/spawning.

The genes with DM CpG include three genes related to collagen metabolism. The first is collagen α-1(VIII), which was downregulated in atrophied muscle. In addition, collagen Prolyl 3-hydroxylase-4 (non-enzymatic) (P3H4) is a part of an enzyme complex that catalyzes the hydroxylation of lysine in collagen α chains and thus is required for normal collagen biosynthesis and its fibrils cross-linking [75]. P3H4 was downregulated in the gravid fish. This list also includes Proline dehydrogenase-1 (PRODHB), an inner mitochondrial membrane flavoprotein related to the electron transport system and ATP production derived from the breakdown of extracellular collagen to sustain intracellular ATP under nutrient stress conditions [76].

The gene list includes glycerophosphodiester phosphodiesterase domain-containing protein-5 (*GDPD5*), which was 20-fold downregulated in atrophying muscle with a hypomethylated CpG in the gene body. A study in mice showed that the expression of a functionally relevant gene named *GDE5* was downregulated in atrophied muscles. The study suggested a muscle atrophy protective role for *GDE5* reduction [77].

Three affected genes are involved in cell membrane functions named Aquaporin-7 and transmembrane protein 151B (*TMEM151B*). Aquaporin-7 is aquaglyceroporin, a component of the muscle cell membrane involved in transporting water molecules and glycerol. Aquaporin-7 mRNA expression is upregulated in the skeletal and cardiac muscles of obese Type II diabetic mice, an insulin resistance model that accelerates muscle protein degradation [78]. We previously observed increased water content in the atrophying muscle cells to fill the mobilized proteins and fat voids, which explains the differential expression and methylation of the Aquaporin gene [24,79]. The other two genes associated with membrane functions are *TMEM151B*, with SNPs associated with lean mass in humans [80], and Ankyrin-1, involved in cell motility and proliferation [81].

A significant outcome of this study is that the previously reported dominant biochemical signs of sexual maturation associated with muscle atrophy, high water content, [24] and reduced glycolysis [23] had relevant genes with congruent differential expression and methylation. Another notable result is that genes described in this section, *PRODHB*, *GDPD5*, and Sestrin, are all involved in activating cells’ survival mode due to the significant loss of muscle mass.

#### 3.4.5. Homeobox Proteins

The study showed 3 DM CpGs in 2 homeobox genes that were downregulated in atrophying muscle; *HOX-C5A* and SIX homeobox 4. These genes are the only genes that showed DE among 51 genes relevant to homeobox proteins exhibiting DM CpGs (discussed above). Downregulation of a homeobox member *HOXA10* was reported during muscle regeneration after damage in mice [82], and hypermethylation of *HOX* genes in myogenic cells was reported to help regulate *HOX* gene expression [83]. In addition, several *HOX* genes were hypermethylated in geriatric versus young human muscle; a negative relationship between DNA methylation and gene expression was observed. Physical activity was reported to help reverse these age-related epigenetic changes [84].

#### 3.4.6. Other Epigenetic Markers

This category includes 11 genes with various or uncharacterized functions. The list includes HSP90ß1A, whose expression was downregulated in starved Atlantic salmon and upregulated during muscle differentiation [85]. The list also includes a molecular chaperone gene family member, T-complex protein-1 subunit epsilon [86]. Other genes with essential functions include cytochrome c1, which encodes a subunit of the cytochrome bc1 complex; CACNA1SB, a subunit of the skeletal muscle calcium channel; and cleavage and polyadenylation specificity factor subunit-1.

## Figures and Tables

**Table 1:** Gene ontology enrichment analysis of DM CpGs categorized in molecular functions (MF) and biological processes (BP).

Source	Term_Name	Term_id	Adj_*p* Value
GO:MF	DNA-binding transcription factor activity, RNA polymerase II-specific	GO:0000981	1.96 × 10^−3^
GO:MF	Metal ion transmembrane transporter activity	GO:0046873	2.24 × 10^−2^
GO:BP	Homophilic cell adhesion via plasma membrane adhesion molecules	GO:0007156	6.60 × 10^−5^
GO:BP	Cell adhesion	GO:0007155	3.18 × 10^−2^
GO:BP	Biological adhesion	GO:0022610	3.18 × 10^−2^
GO:BP	Cation transport	GO:0006812	1.66 × 10^−2^
GO:BP	Metal ion transport	GO:0030001	9.59 × 10^−3^
GO:BP	Cell-cell adhesion	GO:0098609	2.59 × 10^−4^
GO:BP	Cell–cell adhesion via plasma-membrane adhesion molecules	GO:0098742	1.28 × 10^−4^

**Table 2:** DM CpGs and their associated differential gene expression in atrophied muscle of gravid/2N fish compared to sterile/3N fish. Highlighted cells in green and red indicates positive and negative changes. Highlighted TFs are the most common transcription factors.

Chr Position	Gene/Locus	Position to TSS	Hypo/Hyper Meth (2N–3N)	Meth. Diff *p* Value	Exp. Fold Change (2N/3N)	Exp. FDR	Exp/Meth R Value	Gene Annotation	TF
**Apoptosis and epigenetic regulation**									
NC_035082.1_64665435	LOC110526468/Pdcd5	−899	33.3	3.2 × 10^−5^	**−2.10**	7.71 × 10^−2^	−0.441	Programmed cell death protein 5	HES-1 [T01649]; RXR-alpha [T01345]; 3; MYB2 [T02536]; AR [T00040]; USF-1 [T00875]; NF-1 [T00537]; NF-1 [T00539]
NC_035082.1_64665694	LOC110526468/Pdcd6	−640	63.5	3.2 × 10^−5^	**−2.10**	7.71 × 10^−2^	−0.497	Programmed cell death protein 5	c-Ets-1 [T00112]
NC_035078.1_41106515	LOC110492018/AEN	4446	−48.8	7.5 × 10^−6^	**−3.52**	1.01 × 10^−4^	0.434	Apoptosis-enhancing nuclease	E2F-1 [T01542]; C/EBPbeta [T00581]; C/EBPgamma [T00216]; NF-1 [T00535]
NC_035081.1_29419783	LOC110523624/Smyd1	3041	−65.0	4.0 × 10^−7^	**−2.95**	6.10 × 10^−3^	0.459	Histone-lysine N-methyl transferase Smyd1	LyF-1 [T00479]; PTF1-beta [T00701]; myogenin [T00528]; Pax-5 [T00070]
NC_035088.1_13620476	LOC110537077/Cdk2ap1	−586	48.0	1.7 × 10^−5^	**−7.36**	9.39 × 10^−5^	−0.439	Cyclin-dependent kinase 2-associated protein 1	USF1 [T00874]; USF1 [T00874]; c-Myc [T00140]; RXR-beta [T01349]; RXR-beta [T01349]; PIF3 [T04492]; PIF3 [T04492]; SREBP-1c [T01562]; CBF1 [T00080]; CBF1 [T00080]; JunD [T00437]; JunD [T00437]
NC_035089.1_41352275	LOC110486510/Ddx39a	4421	−50.0	9.9 × 10^−6^	**−2.26**	5.57 × 10^−2^	0.625	ATP-dependent RNA helicase DDX39A	XBP-1 [T00902]; E2F-1 [T01542]; f(alpha)-f(epsilon) [T00287]
NC_035089.1_41352276	LOC110486510/Ddx39a	4422	−55.0	5.2 × 10^−6^	**−2.26**	5.57 × 10^−2^	0.264	ATP-dependent RNA helicase DDX39A	XBP-1 [T00902]; E2F-1 [T01542]; f(alpha)-f(epsilon) [T00287]
**Autophagy, glycolysis, collagen metablisim, cell memberane**									
NC_035082.1_53102690	LOC110526208/Atg13	−752	−50.6	1.3 × 10^−5^	**2.50**	7.86 × 10^−2^	−0.674	Autophagy-related protein 13	Pax-2.2 [T03219]; Pax-9a [T03593] Pax-9b [T03594]
NC_035101.1_26128247	LOC110504991/Sesn3	5519	27.6	3.4 × 10^−5^	**3.69**	1.25 × 10^−2^	0.820	Sestrin-3	GAGA factor [T00301]
NC_035093.1_46301679	LOC110494292/Pfkfb3	5567	80.3	6.7 × 10^−16^	**4.33**	2.86 × 10^−3^	0.725	6-phosphofructo-2-kinase	MCB1 [T06035]; MCB2 [T06036]; Pax-6 [T00682]
NC_035094.1_5494483	LOC110495489/Col8a1	8739	−55.6	3.5 × 10^−5^	**−7.93**	1.30 × 10^−4^	0.686	Collagen alpha-1(VIII) chain	NF-E4 [T00560]; Sp1 [T00754]; Sp1 [T00759]
NC_035088.1_66751403/island11919	LOC110538176/P3h4	2242	−54.8	7.1 × 10^−7^	**−3.68**	7.35 × 10^−2^	0.283	Prolyl 3-hydroxylase family member 4 (non-enzymatic)	USF1 [T00874]; USF1 [T00874]; c-Myc [T00140]; PHO4 [T00690]; USF-1 [T00875]; USF-1 [T00875]; HES-1 [T01649]; HES-1 [T01649]
NC_035082.1_40371257	LOC110526004/Prodhb	4547	46.4	6.4 × 10^−6^	**2.74**	1.73 × 10^−2^	0.594	Proline dehydrogenase 1, mitochondrial	Pax-5 [T00070]; YY1 [T04970]; RXR-beta [T01349]; HES-1 [T01649]; AhR [T00018]; AhR [T01795]; JunD [T00437]
NC_035088.1_41298016	LOC110537666/Gdpd5	6026	−47.6	1.2 × 10^−5^	**−20.04**	3.30 × 10^−6^	0.139	Glycerophosphodiester phosphodiesterase domain-containing protein 5	
NC_035093.1_33080772	LOC110494010/Aqp7	9509	29.3	9.4 × 10^−6^	**6.05**	2.32 × 10^−3^	0.358	Aquaporin-7	SXR:RXR-alpha [T05670]; HES-1 [T01649]
NC_035105.1_28018844/island23784	LOC110509954/Tmem151b	5185	30.6	3.3 × 10^−5^	**10.35**	1.65 × 10^−3^	0.639	Transmembrane protein 151B	Pax-5 [T01201]; Nkx2-1 [T00857]; GAL4 [T00302]
NC_035087.1_56212451	LOC110536085/Ank1	5063	−59.5	1.2 × 10^−5^	**−2.44**	6.32 × 10^−2^	0.079	Ankyrin-1	NFI/CTF [T00094]; NF-AT2 [T01945]; PEA3 [T00684]; NF-AT1 [T01948]; NF-AT1 [T01944];NF-AT1 [T00550]; HNF-4alpha1 [T00372]
**Homeobox proteins**									
NC_035092.1_38302815	LOC110492001/Hox-C5a	1862	−47.6	7.8 × 10^−6^	**−6.03**	1.48 × 10^−2^	0.474	Homeobox protein Hox-C5a	c-Myb [T00138]
NC_035092.1_38302823	LOC110492001/Hox-C5a	1870	−45.7	2.6 × 10^−5^	**−6.03**	1.48 × 10^−2^	0.382	Homeobox protein Hox-C5a	Sp1 [T00755]; Nkx2-1 [T00857]
NC_035101.1_61368566	Six4	2175	−50.8	3.6 × 10^−5^	**−3.08**	6.73 × 10^−2^	0.015	SIX homeobox 4	MF3 [T00507]; ENKTF-1 [T00255]; Tll [T00789]; E2F [T00221]; POU4F1(l) [T01877]; Pax-5 [T00070]
**Other epimarkers**									
NC_035084.1_10667325	Hsp90ba	6124	63.5	8.6 × 10^−7^	**2.00**	9.16 × 10^−2^	0.698	Heat shock 90kDa protein 1 beta isoform a	TAF [T00778]
NC_035091.1_7350831	LOC110489569/Ccts	3278	−45.2	3.7 × 10^−6^	**−2.72**	8.21 × 10^−4^	0.297	T-complex protein 1 subunit epsilon	Mad [T04378]; LF-A1 [T00467]; MF3 [T00507]; E2F-1 [T01543]; YY1 [T00865]; XBP-1 [T00902]; YY1 [T00915]; Sp1 [T00754]; Sp1 [T00759]; YY1 [T00278]; ENKTF-1 [T00255]
NC_035084.1_34778859	LOC110529890/PGBD4	923	31.6	1.9 × 10^−6^	**−8.21**	8.85 × 10^−6^	−0.505	PiggyBac transposable element-derived protein 4	HNF-1B [T01950]; Crx [T03461]; POU2F1 [T00643]
NC_035077.1_13381573	LOC110493215/Mast3	4520	33.3	1.2 × 10^−5^	**2.68**	9.8 × 10^−2^	−0.119	Microtubule-associated serine/threonine-protein kinase 3	C/EBP [T01388]; CREMtau [T01309]; CREMtaualpha [T01602]; CREMtau1 [T02108]; CREMtau2 [T02109]
NC_035092.1_36914362	LOC110491948/Cacna1sb	2646	−38.6	2.0 × 10^−6^	**−5.37**	2.96 × 10^−6^	0.858	Dihydropyridine-sensitive L-type skeletal muscle calcium channel subunit alpha-1	MED8 [T03491]; C/EBP [T01388]; C/EBP [T01388]
NC_035092.1_29706361	LOC110491830/Cytc-1	4423	−43.8	3.3 × 10^−5^	**−2.80**	1.46 × 10^−4^	0.123	Cytochrome c1	Mad [T04378]; GA-BF [T00297]
NC_035090.1_23141191	LOC110488198/Cpsf1	8837	−50.0	2.1 × 10^−7^	**−3.83**	1.34 × 10^−2^	0.406	Cleavage and polyadenylation specificity factor subunit 1	Pax-5 [T00070]
NC_035093.1_25737498	LOC110493184/Znfx-1	2285	−29.7	4.8 × 10^−5^	**−2.61**	7.87 × 10^−2^	0.324	NFX1-type zinc finger-containing protein 1	
NC_035097.1_35527983	LOC110500515/Gilgyf1	6282	48.8	1.7 × 10^−5^	**2.74**	7.18 × 10^−2^	0.772	GRB10-interacting GYF protein 1	Mad [T04378]; Pax-6 [T00682]
NC_035095.1_15029930	LOC110497457	4136	47.8	2.0 × 10^−5^	**67.32**	8.65 × 10^−3^	0.637	Vegetative cell wall protein gp1	
NC_035097.1_10910032	LOC110501017	164	51.6	5.3 × 10^−8^	**−4.36**	2.37 × 10^−2^	−0.417	Uncharacterized LOC110501017	HSF1 (long) [T01042]; HSF1 (short) [T02104]; AP-4 [T00036]; myogenin [T00528]; Cutl1 [T02042]; HOXA5 [T00377]; POU1F1a [T00691];PF1 [T04784]

## Data Availability

The sequencing datasets supporting the conclusions of this article Sequence data are available through the Sequence Read Archive (SRA), NCBI accession: PRJNA431930. All datasets generated for this study are included, referred to in the manuscript and/or the Supplementary Files.

## Supplementary Materials

The following supporting information can be downloaded at: https://www.mdpi.com/article/10.3390/genes13071151/s1. Supplementary Table S1—Genes with hypomethylated (0–44%) CGIs marks in the 0-to+2 kb region (shown in Figure 8). Supplementary Table S2—Gene Ontology annotations of genes with hypomethylated (0–44%) CGIs marks in the 0-to+2 kb region (Figure 8). Supplementary Table S3: DM CpGs within −1 to +10 kb flaking TSS. Supplementary Table S4: Silenced genes (RPKM Decile 1) in rainbow trout muscle have more hypermethylated CGIs in the promoter region compared to the most highly expressed genes (Decile 10).
